# Survey data on perceptions of water scarcity and potable reuse from water utility customers in Albuquerque, New Mexico

**DOI:** 10.1016/j.dib.2020.105289

**Published:** 2020-02-13

**Authors:** Lauren N. Distler, Caroline E. Scruggs

**Affiliations:** a Community and Regional Planning Department, School of Architecture and Planning, University of New Mexico, Albuquerque, NM, USA; b Water Resources Program, University of New Mexico, Albuquerque, NM, USA

**Keywords:** Water recycling, Potable reuse, Public opinion, Trust, Water scarcity, Water resources planning, Survey research, Public education and outreach

## Abstract

The data presented in this article were collected using a large-scale public survey distributed through the mail to a random sample of 4000 water utility residential account holders in Albuquerque, New Mexico, USA. The survey collected data on a variety of water-related topics, including water scarcity, climate change, water use at home, perceptions of water sources and water quality, conservation habits, level of acceptance of two potable water reuse scenarios, and level of trust in a variety of entities. The survey also collected demographic data from respondents. Account holders received one of four survey versions, three of which provided different sets of educational material to test different motivations for accepting potable water reuse, and one provided no educational material. The survey was designed and administered using the Tailored Design Method, which involved focus groups, individual debriefing sessions, and a pre-test with members of the sample population to refine the survey instrument, and included a system of five contacts mailed out over a period of several months to maximize response rate. Mail-in and electronic response options were available, and the response rate was 46% (n = 1831). The data were compiled using Survey Monkey and organized using Microsoft Excel and RStudio. The data set featured in this article provides raw survey data plus additional variables created by grouping and consolidating answer options in the raw data. This is the first and most comprehensive set of data known to the authors on public perceptions of water resources and reuse in an arid inland community, and the authors have published open access papers based on this data set, which are linked to this paper. Water managers, planners, engineers, and utilities may be interested in using the data as a point of comparison for their own study on community knowledge of water resources and acceptance of water reuse or in examining the data for relationships not yet explored in the literature.

**Table:** Specifications Table

Subject	Social Sciences – Geography, Planning and Development
Specific subject area	Water Resources Planning and Management
Type of data	Table (.csv format) Supporting materials (codebook and text version of survey instrument)
How data were acquired	The data were acquired through a community survey, which was conducted with approval by the University of New Mexico Institutional Review Board. Instruments: Survey Monkey, Microsoft Excel, and RStudio used to collect, organize, and clean the data set.
Data format	Raw. Additional simplified variables created by consolidating raw data.
Parameters for data collection	Surveys were sent to a random sample of 4,000 water utility account holders in Albuquerque and Bernalillo County, New Mexico, USA. The random sample was selected from the Albuquerque Bernalillo County Water Utility Authority's customer accounts log. The sample was created such that the proportions of account holders living in each city quadrant (NE, SE, SW, NW) were adequately represented. Participants were required to be 18 years of age or older and account holders with the Albuquerque Bernalillo County Water Utility Authority.
Description of data collection	Survey recipients had the option to respond through the mail or online. Those who responded online entered their answers into Survey Monkey. The data for those who opted for the mail-in response were entered manually into Survey Monkey by a member of the research team. All data were then extracted from Survey Monkey in.csv format for cleaning and simplification in Excel and RStudio.
Data source location	City/Town/Region: Albuquerque, New Mexico Country: United States of America
Data accessibility	All data are provided as supplementary material alongside this article.
Related research articles	1) L. N. Distler and C. E. Scruggs, In Press, “Arid Inland Community Survey on Water Knowledge, Trust and Potable Reuse. I: Description of Findings.” *Journal of Water Resources Planning and Management*. DOI:10.1061/(ASCE)WR.1943-5452.0001218. 2) L. N. Distler, C. E. Scruggs, and K. N. Rumsey, In Press, “Arid Inland Community Survey on Water Knowledge, Trust and Potable Reuse. II: Predictive Modeling.” *Journal of Water Resources Planning and Management*. DOI:10.1061/(ASCE)WR.1943-5452.0001219.

**Table:** 

**Value of the Data** • To the authors' knowledge, this is the first large-scale survey conducted in an arid inland community on public water knowledge and perceptions of potable water reuse. Thus, the data will be a useful point of comparison for similar surveys conducted in other regions. • The dataset and associated materials will provide researchers, water utilities and other interested entities with information and methods on conducting a large-scale public survey on water-related topics. • While the authors have published articles related to the data described in this article, these analyses were not exhaustive. Water utilities, planners, engineers, and managers may be interested in other facets of the data that have not yet been analyzed. By making the data set freely available, others can perform further analyses that are useful to the advancement of knowledge on issues related to water reuse in arid inland areas. • With water scarcity becoming a critical issue around the world, more data is needed in additional contexts regarding management of water resources and the implementation of technologies useful in adapting to climate change.

## 1 Data description

The data associated with this article includes three components:

1) *The survey instrument used to collect the data (SurveyInstrument_summarized.pdf)*. The survey instrument contains 26 questions. Nine of these concern demographics, and the others are related to opinions and information about water scarcity, climate change, level of concern with community issues, water habits at home, level of acceptance of two potable reuse scenarios (as well as reasons for support or concern), trust in various institutions, and other water resource topics. A text-only version of the survey is included in the supplementary materials alongside this paper, and the more complete survey instrument that was distributed to ABCWUA account holders is discussed in the next section of this paper.
2) *The raw survey data in.csv file format (SurveyData.csv)*. The survey data file contains 93 variables and data from each of the 1831 respondents. Blanks or non-responses in the dataset are coded as “NA” (i.e., no answer). Additional variables were created by grouping or consolidating categories within each survey question for simpler analysis. These variables are listed in the last columns of the file, as outlined by the codebook.
3) *The codebook (Codebook.pdf)*. The codebook explains each of the 93 variables included in the survey data file. The codebook concisely lists how each survey question and response option is numerically coded in the raw data and should be used as a guide for navigating the data set.

## 2 Experimental design, materials, and methods

We collaborated on a community survey with the Albuquerque Bernalillo County Water Utility Authority (ABCWUA), the sole provider of water and wastewater services to the greater Albuquerque metropolitan area, serving over 600,000 water users. Similar to previous research, the survey asked ABCWUA account holders about their water knowledge, water habits, opinions on two potable reuse scenarios, level of trust in institutions, a variety of water- and climate change-related topics, and demographic information [[1], [2], [3]]. There were four versions of the survey, differing only by page five: Version 1 was the control and contained no additional material, while each of the other three versions contained a different set of educational materials on page five, since certain types of educational materials are thought to influence perceptions and opinions related to water reuse [4]. As outlined in the codebook and survey instrument, Versions 2, 3, and 4 provided information on “Water Sources and Reliable Supplies”, “Environmental Benefits of Water Reuse”, and “The Urban Water Cycle”, respectively. The complete survey instrument that was distributed to ABCWUA account holders is linked to our other publications associated with this data, which were published open access [5,6].

The steps used to design and implement our survey included a number of focus groups and debriefing sessions involving individual members of our sample population [7,8]. Eight 90-minute focus groups, each with 7-10 participants, allowed us to test prototype survey questions and identify and develop other content to include in the survey [9]. The focus groups were conducted at familiar and accessible locations throughout the community. Participants were required to be at least 18 years of age and ABCWUA customers. Part-way through and following completion of the focus groups, we tested the draft survey on 12 individual members of the sample population in a series of one-on-one survey debriefing sessions. During each session, the participant was asked to complete the survey while thinking out loud with a researcher present. Debriefings allowed researchers to ensure that survey questions and materials were being interpreted and understood correctly and determine the time needed to complete the survey. [7,8]. We refined the survey instrument throughout the eight focus groups and 12 debriefing sessions. The focus groups were conducted in July, October, and November, and the debriefing sessions were held in August, October, and November of 2016.

Four thousand accounts were randomly selected from over 180,000 residential accounts contained in the ABCWUA customer accounts log. The log provided a mailing address for each customer, among other variables (e.g., Census tract, ZIP code). Customer names were immediately deleted from the sample file and addresses were deleted following the conclusion of data analyses. Each potential respondent was assigned a unique random code as an identifier, which provided an anonymous way to track responses. The customer accounts log also provided information on the quadrant of the city in which each customer resided. The proportion of the sample in each quadrant was checked against the proportions in the customer accounts log to ensure that the proportions of the sample and the population matched (within 1%). We conducted the survey by mail since we only had customers' physical addresses as a means of contacting them. However, the mailed invitation to complete the survey included an option to complete it online through Survey Monkey.

To verify our survey instrument, we pretested it on a random sample of 200 water utility customers, mimicking the actual, larger survey sample population. The pretest also allowed us to understand the likely response rate for the larger survey and the soundness of our administration procedures [8]. Based on the results of the pretest, we finalized our survey instrument and administration process. We sent the survey to a random sample of 4,000 ABCWUA account holders, which were evenly divided across the four survey versions, with 1000 account holders receiving each version. Following procedures recommended in Dillman et al.’s Tailored Design Method [7], we administered a system of five contacts over a time period of eight weeks, from April through June of 2017. We closed the survey to additional responses on September 5, 2017.

Mail-in responses were manually entered into Survey Monkey. The compiled survey responses in Survey Monkey were exported to.csv format, and Microsoft Excel and RStudio were used to organize and clean the data.

## Funding

This work was supported by the National Science Foundation [grant number 1345169]. Funders were not involved in the study design, data analysis, or dissemination of results.

## References

[1] Macpherson L., Slovic P. (2011). Talking about Water: Vocabulary and Images that Support Informed Decisions about Water Recycling and Desalination.

[2] Millan M., Tennyson P.A., Snyder S.A. (2015). Model Communication Plans for Increasing Awareness and Fostering Acceptance of Direct Potable Reuse.

[3] Macpherson L., Snyder S.A. (2013). Downstream: Context, Understanding, Acceptance: Effect of Prior Knowledge of Unplanned Potable Reuse on the Acceptance of Planned Potable Reuse.

[4] United States Environmental Protection Agency (2012). Guidelines for Water Reuse.

[5] Distler L.N., Scruggs C.E. (2020). Arid inland community survey on water knowledge, trust and potable reuse. I: description of findings. J. Water Resour. Plann. Manag..

[6] Distler L.N., Scruggs C.E., Rumsey K.N. (2020). Arid inland community survey on water knowledge, trust and potable reuse. II: predictive modeling. J. Water Resour. Plann. Manag..

[7] Dillman D.A., Smyth J.D., Christian L.M. (2014). Internet, Phone, Mail, and Mixed-Mode Surveys: the Tailored Design Method.

[8] Thacher J., Marsee M., Pitts H., Hansen J., Chermak J., Thomson B. (2011). Assessing Customer Preferences and Willingness to Pay: a Handbook for Water Utilities.

[9] Krueger R.A., Casey M.A. (2000). Focus Groups: a Practical Guide for Applied Research.

