# Supplementary material for: Survey data on perceptions of water scarcity and potable reuse from water utility customers in Albuquerque, New Mexico
Source: Data Brief. 2020 Feb 13;29:105289. doi: 10.1016/j.dib.2020.105289 (PMC7056618; doi:10.1016/j.dib.2020.105289)
Supplement: Multimedia component 1 [file mmc1.pdf]

Codebook - Main Survey Data

Lauren N Distler

January 1 2019

Key

VARIABLE NAME
Variable description
Data type (Continuous, Discrete, Nominal, Ordinal)
Item Value Description

Respondent Information

CODE
Random unique identification number
Nominal
Range: 100123-555555

VERSION
Survey version (information treatment)
Nominal
1. CONTROL (NO INFORMATION)
2. WATER SOURCES & RELIABLE SUPPLIES
3. ENVIRONMENTAL BENEFITS OF WATER REUSE
4. THE URBAN WATER CYCLE

MODE
Survey mode used
Nominal
"MAIL", "ONLINE".

ZIP
5 digit zipcode
Nominal
Range: 87102-87123

QUAD
Quadrant of city
Nominal
"NW", "NE", "SE", "SW".

Raw Survey Data (Non-responses coded as “NA” in .csv)

Question 1: How concerned are you about the following issues in Albuquerque/Bernalillo County? *(Circle your level of concern for each issue)*

```
DROUGHT_CONCERN
Level of concern with drought/water shortage
Ordinal
1. Not at all concerned
2. Slightly concerned
3. Moderately concerned
4. Very concerned
5. Extremely concerned

EDUCATION_CONCERN
Level of concern with quality of public education in local schools
Ordinal
1. Not at all concerned
2. Slightly concerned
3. Moderately concerned
4. Very concerned
5. Extremely concerned

POPULATION_CONCERN
Level of concern with population growth and development
Ordinal
1. Not at all concerned
2. Slightly concerned
3. Moderately concerned
4. Very concerned
5. Extremely concerned

JOBS_CONCERN
Level of concern with jobs and local economy
Ordinal
1. Not at all concerned
2. Slightly concerned
3. Moderately concerned
4. Very concerned
5. Extremely concerned

CRIME_CONCERN
Level of concern with crime rate
Ordinal
1. Not at all concerned
2. Slightly concerned
3. Moderately concerned
4. Very concerned
5. Extremely concerned
```

TAXES_CONCERN
Level of concern with amount paid in local taxes
Ordinal
1. Not at all concerned
2. Slightly concerned
3. Moderately concerned
4. Very concerned
5. Extremely concerned

WATERQUAL_CONCERN
Level of concern with local drinking water quality
Ordinal
1. Not at all concerned
2. Slightly concerned
3. Moderately concerned
4. Very concerned
5. Extremely concerned

WATERBILL_CONCERN
Level of concern with amount paid on water bill
Ordinal
1. Not at all concerned
2. Slightly concerned
3. Moderately concerned
4. Very concerned
5. Extremely concerned

Question 2: In your opinion, do you think water is a limited resource in Albuquerque? *(Check one)*

SCARCITY
Perception of water scarcity in area
Nominal
Y. Yes
N. No
DK. I dont know

Question 3: From what source or sources does the Albuquerque Bernalillo County Water Utility Authority (ABCWUA) get the water it serves to customers? *(Check all that apply)*

SOURCE_SW
Indicated surface water as a source for ABQ
Nominal
0. NO
1. YES

SOURCE_GW
Indicated ground water as a source for ABQ
Nominal
0. NO
1. YES

SOURCE_DK
Indicated that they do not know the source for ABQ
Nominal
0. NO
1. YES

SOURCE_OTHER
Indicated other as a source for ABQ
Nominal
0. NO
1. YES

Question 4: Do you believe that the impact of climate change on the water cycle will make it more difficult for ABCWUA to meet our community's water needs in the next 10 to 40 years? *(Check one)*

CLIMATE
Belief in climate change
Nominal
Y. Yes
N. No
DK. I dont know

Question 5: Do you believe that bottled water is safer (higher quality) than Albuquerque tap water? *(Check one)*

BOTTLED_WATER
Percieves bottled water to be safer
Nominal
Y. Yes
N. No
DK. I dont know

Question 6: What type of water do you most often drink at home? *(Check one)*

HOME_WATER
Which type of water do you most often drink at home?
Nominal
1. City tap water
2. City tap water filtered at home
3. Water from private well
4. Bottled water
5. Purfied water from dispenser at store
6. Other

Question 7: Which of the following [water conservation measures] are you currently doing at home? *(Check all that apply)*

CONSERVE_XERI
Xeriscaped land/yard
Nominal
0. NO
1. YES

CONSERVE_YARD
Do not water land/yard
Nominal
0. NO
1. YES

CONSERVE_FIXTURES
Use water saving fixtures (e.g.,faucets, toilets)
Nominal
0. NO
1. YES

CONSERVE_APPLIANCES
Use water efficient appliances (e.g.,dishwasher,washing machine)
Nominal
0. NO
1. YES

CONSERVE_RAINWATER
Practice rainwater harvesting (e.g.,rain barrel)
Nominal
0. NO
1. YES

CONSERVE_SIMPLE
Use simple conservation measures (e.g., turning off water when brushing teeth)
Nominal
0. NO
1. YES

CONSERVE_NONE
Indicated that they do not conserve (none of the above)
Nominal
0. NO
1. YES

Question 8: Generally speaking, how aware are you of water scarcity issues in New Mexico? *(Circle one)*

```
SCARCITY_AWARE
Level of awareness
Ordinal
1. Not at all aware
2. Slightly aware
3. Moderately aware
4. Very aware
5. Extremely aware
```

Question 9: Are you aware of the concept of purifying wastewater and reusing it for drinking water? *(Check one)*

```
REUSE_AWARE
Prior awareness of potable reuse
Nominal
Y. Yes
N. No
```

Question 10: How willing would you be to drink the city tap water in Community A? *(Circle one)*

```
DPR_WILL
Level of willingness - Direct Potable Reuse
Ordinal
1. Refuse to drink
2. Prefer to avoid
3. Neutral
4. Generally OK
5. Very willing to drink
```

Question 11: For what reason(s) would you be willing to drink the city tap water in Community A? *(Check all that apply)*

```
DPR_UNWILL
Indicated that they would NOT be willing to accept DPR, no reasons to accept
0. NO
1. YES

DPR_WILL_SCARCITY
Indicated that they would be willing to accept DPR due to water shortage, drought, or
limited supply.
0. NO
1. YES
```

DPR_WILL_WASTE
Indicated that they would be willing to accept DPR due to reduction in waste, efficient use of resources.
0. NO
1. YES

DPR_WILL_SAFE
Indicated that they would be willing to accept DPR due to purified water being safe to drink and safely consumed in other US cities.
0. NO
1. YES

DPR_WILL_TRUST
Indicated that they would be willing to accept DPR due to trust in purification technologies
0. NO
1. YES

DPR_WILL_OTHER
Indicated that they would be willing to accept DPR due to another reason (option to write-in)
0. NO
1. YES

Question 12. What concern(s) might you have about drinking the city tap water in Community A? *(Check all that apply)*

DPR_NOCONCERN
Indicated that they had no concerns with DPR
0. NO
1. YES

DPR_CONC_TRUST
Indicated concern with DPR due to lack of trust in the purification technologies
0. NO
1. YES

DPR_CONC_SAFE
Indicated concern with DPR due to lack of confidence in the safety of the water/health concerns
0. NO
1. YES

DPR_CONC_GOVT
Indicated concern with DPR due to lack of trust in the government/water utility
0. NO
1. YES

DPR_CONC_TASTE
Indicated concern with DPR due to expectation of bad taste/smell or discoloration of the water
0. NO
1. YES

DPR_CONC_OTHER
Indicated concern with DPR for another reason (option to write-in)
0. NO
1. YES

Question 13: How willing would you be to drink the city tap water in Community B? *(Circle one)*

IPR_WILL
Level of willingness - Indirect Potable Reuse
Ordinal
1. Refuse to drink
2. Prefer to avoid
3. Neutral
4. Generally OK
5. Very willing to drink

Question 14: For what reason(s) would you be willing to drink the city tap water in Community B? *(Check all that apply)*

IPR_UNWILL
Indicated that they would NOT be willing to accept IPR, no reasons to accept
0. NO
1. YES

IPR_WILL_SCARCITY
Indicated that they would be willing to accept IPR due to water shortage, drought, or limited supply.
0. NO
1. YES

IPR_WILL_WASTE
Indicated that they would be willing to accept IPR due to reduction in waste, efficient use of resources.
0. NO
1. YES

IPR_WILL_SAFE
Indicated that they would be willing to accept IPR due to purified water being safe to drink and safely consumed in other US cities.
0. NO
1. YES

IPR_WILL_TRUST
Indicated that they would be willing to accept IPR due to trust in purification technologies
0. NO
1. YES

IPR_WILL_ENV
Indicated that they would be willing to accept IPR due to the water passing through the environment before it is treated and used again
0. NO
1. YES

IPR_WILL_OTHER
Indicated that they would be willing to accept IPR due to another reason (option to write-in)
0. NO
1. YES

Question 15. What concern(s) might you have about drinking the city tap water in Community B? *(Check all that apply)*

IPR_NOCONCERN
Indicated that they had no concerns with IPR
0. NO
1. YES

IPR_CONC_TRUST
Indicated concern with IPR due to lack of trust in the purification technologies
0. NO
1. YES

IPR_CONC_SAFE
Indicated concern with IPR due to lack of confidence in the safety of the water/health concerns
0. NO
1. YES

IPR_CONC_GOVT
Indicated concern with IPR due to lack of trust in the government/water utility
0. NO
1. YES

IPR_CONC_TASTE
Indicated concern with IPR due to expectation of bad taste/smell or discoloration of the water
0. NO
1. YES

IPR_CONC_OTHER
Indicated concern with IPR for another reason (option to write-in)
0. NO
1. YES

Question 16. Based on the information provided, with which of the following statements do you most agree? *(Check one)*

```
MORE_WILL
Which type of reuse is more acceptable to you?
Nominal
1. DPR
2. IPR
3. Both are equally acceptable
4. Neither is acceptable
```

Question 17. Please indicate how much you would trust each of the following entities to provide you with accurate information on water reuse and the safety of drinking water reuse.*(Circle the appropriate answer for each)*

```
TRUST_ABCWUA
Level of trust in local water agency
Ordinal
1. Mostly distrust
2. Somewhat distrust
3. Neutral
4. Somewhat trust
5. Mostly trust
```

```
TRUST_LOCALGOV
Level of trust in elected local officials
Ordinal
1. Mostly distrust
2. Somewhat distrust
3. Neutral
4. Somewhat trust
5. Mostly trust
```

```
TRUST_REGULATORS
Level of trust in state and federal regulators
Ordinal
1. Mostly distrust
2. Somewhat distrust
3. Neutral
4. Somewhat trust
5. Mostly trust
```

```
TRUST_RESEARCHERS
Level of trust in academic researchers
Ordinal
1. Mostly distrust
2. Somewhat distrust
3. Neutral
4. Somewhat trust
5. Mostly trust
```

TRUST_PUBHEALTH
Level of trust in public health professionals
Ordinal
1. Mostly distrust
2. Somewhat distrust
3. Neutral
4. Somewhat trust
5. Mostly trust

TRUST_LOCALMEDIA
Level of trust in local media
Ordinal
1. Mostly distrust
2. Somewhat distrust
3. Neutral
4. Somewhat trust
5. Mostly trust

TRUST_NPOS
Level of trust in environmental nonprofit organizations
Ordinal
1. Mostly distrust
2. Somewhat distrust
3. Neutral
4. Somewhat trust
5. Mostly trust

TRUST_FAMILY
Level of trust in friends and family
Ordinal
1. Mostly distrust
2. Somewhat distrust
3. Neutral
4. Somewhat trust
5. Mostly trust

Demographic Information (Questions 18-22)

AGE
Discrete
Range: 17-98

GENDER
Nominal
F. Female
M. Male
O. Other

CHILDREN
Do you have children under the age of 18 living in your household?
Nominal
Y. Yes
N. No

NM_NATIVE
Have you lived in New Mexico for most of your life?
Nominal
Y. Yes
N. No

ETHNICITY
Are you of Spanish/Hispanic/Latino ethnicity?
Nominal
Y. Yes
N. No

Question 23. The previous question dealt with ethnicity while this one deals with race. Please check the race(s) you consider yourself to be. These categories are the standard categories used by the Census Bureau.*(Check all that apply)*

RACE_WHITE
Identifies as White
Nominal
0. NO
1. YES

RACE_BLACK
Identifies as Black or African American
Nominal
0. NO
1. YES

RACE_AI
Identifies as American Indian or Alaska Native
Nominal
0. NO
1. YES

RACE_ASIAN
Identifies as Asian
Nominal
0. NO
1. YES

RACE_PI
Identifies as Pacific Islander
Nominal
0. NO
1. YES

RACE_OTHER
Identifies as another race
Nominal
0. NO
1. YES

Demographic Information (Questions 24-26)

EDUCATION
What is the highest degree or level of education you have completed?
Ordinal
1. Less than highschool
2. Completed some highschool
3. High school graduate/ GED
4. Completed some college (no degree)
5. Technical or associate degree or specialized certificate
6. Bachelors degree (BA, BS)
7. Masters degree (MA, MS, MBA)
8. Doctorate/Professional degree (PhD, JD, EdD, MD, DDS)

POLITICAL
With which political party do you primarily identify?
Nominal

Note: Independent and No Affiliation categories were combined from original survey response options

DEM. Democrat
REP. Republican
IND/NONE. Independent/No Affiliation
LIB. Libertarian
GRE. Green

INCOME
Which range best describes your total household income before taxes in 2016?
Ordinal
1. Less than $14,999
2. $15,000 - $24,999
3. $25,000 - $34,999
4. $35,000 - $49,999
5. $50,000 - $74,999
6. $75,000 - $99,999
7. $100,000 - $149,999
8. $150,000 - $199,999
9. $200,000 or more

Additional variables created using raw data for simpler analysis and/or display

DPR and IPR willingness variables collapsed from five into three categories

DPR_WILL_3
Level of willingness - Direct Potable Reuse
Ordinal
"Unwilling", "Neutral", "Willing".

IPR_WILL_3
Level of willingness - Indirect Potable Reuse
Ordinal
"Unwilling", "Neutral", "Willing".

Trust variables collapsed from five into three categories

TRUST_ABCWUA_F
Level of trust in ABCWUA
Ordinal
"Distrust", "Neutral", "Trust".

TRUST_LOCALGOV_F
Level of trust in elected local officials
Ordinal
"Distrust", "Neutral", "Trust".

TRUST_REGULATORS_F
Level of trust in state and federal regulators
Ordinal
"Distrust", "Neutral", "Trust".

TRUST_RESEARCHERS_F
Level of trust in academic researchers
Ordinal
"Distrust", "Neutral", "Trust".

TRUST_PUBHEALTH_F
Level of trust in public health professionals
Ordinal
"Distrust", "Neutral", "Trust".

TRUST_LOCALMEDIA_F
Level of trust in local media
Ordinal
"Distrust", "Neutral", "Trust".

TRUST_NPOS_F
Level of trust in environmental nonprofit organizations
Ordinal
"Distrust", "Neutral", "Trust".

TRUST_FAMILY_F
Level of trust in friends and family
Ordinal
"Distrust", "Neutral", "Trust".

Grouped demographic variables

RACE
Race variables from raw survey data,combined into single variable
Nominal
W. White
B. Black
AI. American Indian
A. Asian
PI. Pacific Islander
O. Other
MIX. Combination of 2 or more

EDUCATION_LEVEL
Original eight education categories collapsed to four
Ordinal
"High school degree or less", "Some college", "College degree", "Advanced degree".

POLITICAL_GROUPED
Green and Libertarian categories combined into "Other"
Nominal
DEM. Democrat
REP. Republican
IND/NONE. Independent/No Affiliation
OTH. Other
