# Supplementary material for: Survey data on perceptions of water scarcity and potable reuse from water utility customers in Albuquerque, New Mexico
Source: Data Brief. 2020 Feb 13;29:105289. doi: 10.1016/j.dib.2020.105289 (PMC7056618; doi:10.1016/j.dib.2020.105289)
Supplement: Multimedia component 2 [file mmc2.pdf]

Survey Instrument (summarized questions)

Question 1. How concerned are you about the following issues in Albuquerque/Bernalillo County? Circle your level of concern for each issue.

1 – Not at all concerned | 2 – Slightly concerned | 3 – Moderately concerned | 4 – Very concerned | 5 – Extremely concerned

Drought/ Water Shortage	1	2	3	4	5
Quality of Public Education in Local Schools	1	2	3	4	5
Population Growth and Development	1	2	3	4	5
Jobs and the Local Economy	1	2	3	4	5
Crime Rate	1	2	3	4	5
Amount Paid in Local Taxes	1	2	3	4	5
Local Drinking Water Quality	1	2	3	4	5
Amount Paid on Water Bill	1	2	3	4	5

Question 2. In your opinion, do you think water is a limited resource in Albuquerque? Check one.

- Yes
- No
- I don't know

Question 3. From what source or sources does the Albuquerque Bernalillo County Water Utility Authority (ABCWUA) get the water it serves to customers? Check all that apply.

- River
- Groundwater aquifer (e.g., well water)
- I don't know
- Other

Question 4. Do you believe that the impact of climate change on the water cycle will make it more difficult for ABCWUA to meet our community's water needs in the next 10 to 40 years? Check one.

- Yes
- No
- I don't know

Question 5. Do you believe that bottled water is safer (higher quality) than Albuquerque tap water? Check one.

- Yes
- No
- I don't know

Question 6. What type of water do you most often drink at home? Check one.

- City tap water
- City tap water filtered at home (e.g., sink, pitcher, or fridge units)
- Water from a private well
- Bottled water (e.g., 12 or 16 oz bottles)
- Purified water from dispenser at a store (e.g., 3 or 5 gal containers)
- Other

Question 7. Which of the following are you currently doing at home? Check all that apply.

- Xeriscaped land/yard
- I don't water my land/yard
- Water saving fixtures (e.g., faucets, toilets)
- Water efficient appliances (e.g., dishwasher, washing machine)
- Rainwater harvesting (e.g., rain barrel)
- Simple conservation measures (e.g., turning off water when brushing teeth)
- None of the above

Question 8. Generally speaking, how aware are you of water scarcity issues in New Mexico? Circle one.

1	2	3	4	5
Not at all aware	Slightly aware	Moderately aware	Very aware	Extremely aware

Question 9. Are you aware of the concept of purifying wastewater and reusing it for drinking water? Check one.

- o Yes
- o No

Question 10. How willing would you be to drink the city tap water in Community A? Circle one.

1	2	3	4	5
Refuse to drink	Prefer to avoid	Neutral	Generally OK	Very Willing to Drink

Question 11. For what reason(s) would you be willing to drink the city tap water in Community A? Check all that apply.

- ☐ Not applicable - I would **not** be willing to drink the water
- ☐ Water shortage, drought, and limited supply
- ☐ Reduces waste; efficient use of resources
- ☐ Purified water is safe to drink and is safely consumed in other US cities
- ☐ I trust the purification technologies
- ☐ Other:________________________

Question 12. What concern(s) might you have about drinking the city tap water in Community A? Check all that apply.

- ☐ No concerns
- ☐ I don't trust the purification technologies
- ☐ I'm not confident the water is safe; health concerns
- ☐ I don't trust the government or water utility
- ☐ I would expect a bad taste/smell or discoloration of the water
- ☐ Other:___________________________

Question 13. How willing would you be to drink the city tap water in Community B? Circle one.

1	2	3	4	5
Refuse to drink	Prefer to avoid	Neutral	Generally OK	Very Willing to Drink

Question 14. For what reason(s) would you be willing to drink the city tap water in Community B? Check all that apply.

- ☐ Not applicable - I would **not** be willing to drink the water
- ☐ Water shortage, drought, and limited supply
- ☐ Reduces waste; efficient use of resources
- ☐ Purified water is safe to drink and is safely consumed in other US cities
- ☐ I trust the purification technologies
- ☐ The water passes through the environment before it is treated and used again
- ☐ Other:________________________

Question 15. What concern(s) might you have about drinking the city tap water in Community B? Check all that apply.

- ☐ No concerns
- ☐ I don't trust the purification technologies
- ☐ I'm not confident the water is safe; health concerns
- ☐ I don't trust the government or water utility
- ☐ I would expect a bad taste/smell or discoloration of the water
- ☐ Other:________________________

Question 16. Based on the information provided, with which of the following statements do you most agree? Check one.

- o I'm more willing to accept Direct Drinking Water Reuse
- o I'm more willing to accept Indirect Drinking Water Reuse
- o Both types of reuse are equally acceptable to me
- o Neither type of reuse is acceptable to me

Question 17. Please indicate how much you would trust each of the following entities to provide you with accurate information on water reuse and the safety of drinking water reuse. Circle the appropriate answer for each.

	1 – Mostly distrust	2 – Somewhat distrust	3 - Neutral	4 – Somewhat trust	5- Mostly trust
Local Water Agency	1	2	3	4	5
Elected Local Officials	1	2	3	4	5
State and Federal Regulators (e.g., NMED, EPA)	1	2	3	4	5
Academic Researchers (e.g., UNM Professors)	1	2	3	4	5
Public Health Professionals (e.g., NM Dept of Health)	1	2	3	4	5
Local Media	1	2	3	4	5
Environmental Nonprofit Organizations	1	2	3	4	5
Friends and Family Members	1	2	3	4	5

Question 18. What is your age?

☐☐ Years

Question 19. What is your gender? Check one.

- o Male
- o Female
- o Other

Question 20. Do you have children younger than 18 years old living in your household? Check one.

- o Yes
- o No

Question 21. Have you lived in New Mexico for most of your life? Check one.

- o Yes
- o No

Question 22. Are you of Spanish/Hispanic/Latino ethnicity? Check one.

- o Yes
- o No

Question 23. The previous question dealt with ethnicity while this one deals with race. Please check the race(s) you consider yourself to be. These categories are the standard categories used by the Census Bureau. Check all that apply.

- ☐ White
- ☐ Black or African American
- ☐ American Indian or Alaska Native
- ☐ Asian
- ☐ Pacific Islander
- ☐ Other

Question 24. What is the highest degree or level of education you have completed? Check one.

- o Less than high school
- o Completed some high school
- o High school graduate/ GED
- o Completed some college (no degree)
- o Technical or Associate degree or Specialized Certificate
- o Bachelor's degree (BA, BS)
- o Master’s degree (MA, MS, MBA)
- o Doctorate/Professional degree (PhD, JD, EdD, MD, DDS)

Question 25. With which political party do you primarily identify? Check one.

- o Democrat
- o Republican

- Independent
- Libertarian
- Green
- No Affiliation

Question 26. Which range best describes your total household income before taxes in 2016? Check one.

- Less than $14,999
- $15,000 to $24,999
- $25,000 to $34,999
- $35,000 to $49,999
- $50,000 to $74,999
- $75,000 to $99,999
- $100,000 to $149,999
- $150,000 to $199,999
- $200,000 or more
